# *Notes from the Field:* Maximizing Tuberculosis Testing After a School-Based Exposure — Lake County, Illinois, 2025

**DOI:** 10.15585/mmwr.mm7504a2

**Published:** 2026-01-29

**Authors:** Amy Zun, Nicolette Meyer, Denise Flores, Tom Mohr, Patricia Aguirre, Tania Rios, Sunu Mathew, Monica Bates, Gabriela Ocampo, Patricia Brady, Patricia Corn, Emily Young, Bree Kingshott, Sara Zamor, Lisa Kritz, Sana Shireen Ahmed

**Affiliations:** 1 Lake County Health Department and Community Health Center, Waukegan, Illinois.

**Summary**

**What is already known about this topic?**

Identifying and testing contacts of persons with infectious tuberculosis (TB) are crucial to detecting cases and preventing transmission and progression of latent tuberculosis infection (LTBI).

**What is added by this report?**

In March 2025, a case of pulmonary TB was reported to the Lake County Health Department and Community Health Center (LCHD) in Illinois; the patient had been present at a Lake County school while infectious. School-guided outreach led to the implementation of communication strategies adapted to community needs. Among 155 school contacts, LCHD examined 143 (92%), including 128 persons who completed two rounds of testing (including one case of LTBI diagnosed during the second event), eight who were tested for the first time during the second event, and seven who received a diagnosis of LTBI during the first testing event. No cases of TB were detected.

**What are the implications for public health practice?**

Close contact investigations identify persons at risk for TB and prevent progression of LTBI when contacts are treated. A flexible approach that facilitates trust and cooperation with the community can support successful testing of numerous contacts.

On March 18, 2025, a case of pulmonary tuberculosis (TB) was reported to the Lake County Health Department and Community Health Center (LCHD) in Illinois. The patient had been present at a Lake County school from November 6, 2024, through March 17, 2025, while infectious. In consultation with the school, LCHD organized testing to identify TB cases and prevent progression of latent TB infection (LTBI) for exposed persons. Among 155 school contacts, LCHD examined 143 (92%), including 128 persons who completed two rounds of testing (including one case of LTBI diagnosed during the second event), eight who were tested for the first time during the second event, and seven who received a diagnosis of LTBI during the first testing event. The school’s awareness of community challenges, including low health literacy and mistrust of government institutions, helped guide LCHD’s response. This activity was reviewed by the Illinois Department of Public Health and was considered a public health practice activity conducted consistent with applicable state law*; institutional review board review was not required.

## Investigation and Outcomes

### Community Characteristics

During 2024, Lake County’s TB incidence (2.5 cases per 100,000 population) was lower than that in Illinois (2.7) and the United States (3.0) overall. The affected school is in a city which is a HealthResourcesandServicesAdministration–designatedmedicallyunderservedarea. Overall, 30.9% of the population are foreign-born persons, 17.3% are living in poverty, and 15.5% do not have health insurance. Although 94% of households have computers, 14% lack internet access (*1*). During the COVID-19 pandemic, shifting guidance, limited bilingual communication, and resource differences exacerbated public mistrust in government institutions, undermining confidence in public health (*2*).

### Close Contact Identification and Selection of Testing Venue

After detection of the index case, the LCHD investigation identified 166 close contacts,^†^ including 156 school contacts (148 students and eight staff members), four household contacts, and six outpatient health care worker contacts (Figure). LCHD collaborated with the school to prioritize clear communication with school-affiliated families, identify an appropriate testing venue, and facilitate testing of identified close contacts.

**FIGURE:**
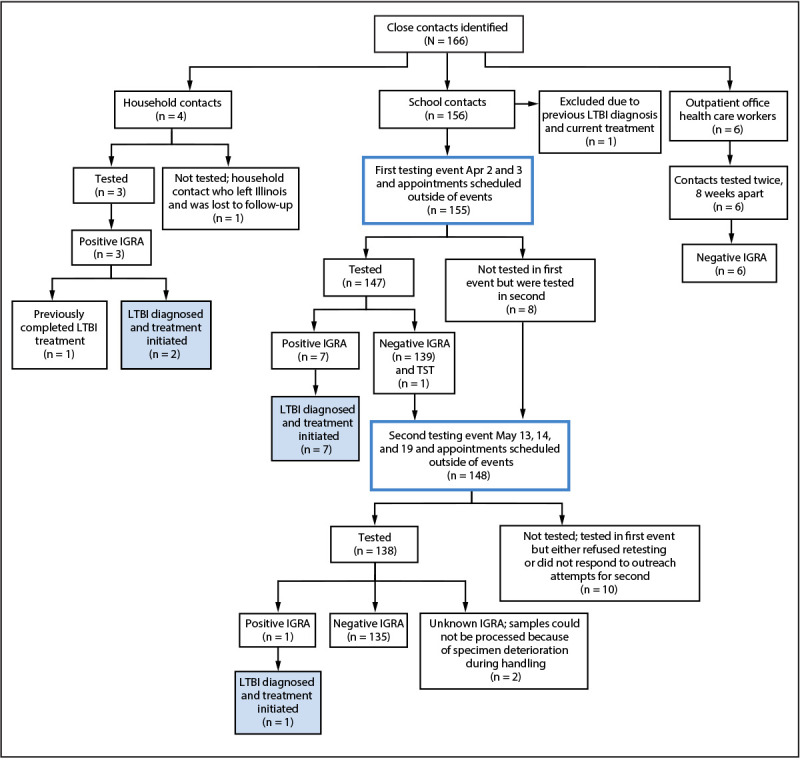
Tuberculosis contact identification, testing, and test results after a school-based exposure — Lake County, Illinois, March 18–July 24, 2025 **Abbreviations**: IGRA = interferon-gamma release assay; LTBI = latent tuberculosis infection; TST = tuberculin skin test.

LCHD initially planned to conduct TB symptom screening and testing at the school; however, based on the school’s insight into families’ concerns about stigma and privacy, as well as possible panic regarding potential TB transmission in the event of a visible health department presence at the school (such as LCHD staff members wearing respirators, the presence of health department vehicles at the school, and TB testing), testing outside the school was considered more likely to encourage testing. Therefore, LCHD was chosen as the screening and testing venue. Community response to emails requesting scheduling of testing at the LCHD was low; however, when LCHD implemented the school’s recommendation to contact families via telephone or text, almost all school contacts were scheduled for testing. To accommodate families’ schedules, the TB clinic also facilitated appointments for testing outside of testing events.

### TB Testing of Household Contacts and Health Care Workers

Among the four household contacts, one left Illinois before testing could be scheduled and could not be located. The other three household contacts received a diagnosis of LTBI after clinical evaluation for symptoms, receipt of positive interferon-gamma release assay (IGRA) test results, and normal chest radiography findings. One had previously completed treatment for LTBI, and the other two were started on LTBI treatment.^§^ All six health care workers were tested through their employer, and all received negative IGRA results.

### TB Testing Events for School Contacts

LCHD organized two testing events for school contacts. The goal of the first event was to provide testing for all contacts. The second event was to provide testing for any contacts who missed the first event and subsequent testing for those who initially received a negative test result, according to CDC recommendations^¶^ (*3*).

**First testing event.** IGRA testing occurred April 2–3. Among 156 identified school contacts, one was receiving LTBI treatment, leaving 155 who needed testing. Among these contacts, 147 (95%) received testing, including 140 who received either a negative IGRA test result (139) or a negative tuberculin skin test (one). Seven contacts received a positive IGRA result and began LTBI treatment after diagnosis by a health care provider.**

**Second testing event.** The event occurred over 3 days (May 13, 14, and 19). LCHD sought to administer testing to 140 school contacts who had received a negative test result and eight contacts who had not received testing during the first event. Among these 148 persons, 136 (92%) received testing, 135 of whom received a negative IGRA test result, and one of whom received a positive test result and a diagnosis of LTBI.

**Combined testing event results.** Overall, 143 of 155 contacts received test results ≥8 weeks after exposure, including 135 who received a negative test result in the second event and the eight persons who received a diagnosis of LTBI from both events. The remaining 12 of the 155 school contacts did not receive test results ≥8 weeks after exposure; two specimens could not be processed because of deterioration, and ten eligible contacts refused retesting or did not respond to outreach.

## Preliminary Conclusions

With successful examination of 143 (92%) of 155 school contacts eligible for testing, LCHD surpassed CDC’s 90% TB contact examination performance target (*4*). No cases of TB disease were identified. Eight (5.2%) contacts received a positive IGRA test result, including seven identified during the first testing event and one during the second; these persons received diagnoses of LTBI and began LTBI treatment.

The success in testing school contacts relied on identification of barriers to parental engagement, flexibility, persistent and respectful outreach, and transparent communication (*5*). LCHD considered the school’s insights and built trust by addressing concerns through bilingual communication, oral education, and hosting testing at LCHD. Flexible testing hours accommodated families’ schedules. These strategies enabled LCHD to foster cooperation, highlighting the value of stakeholder collaboration and adaptability in public health activities.

## Footnotes

*
Title77Ill.Adm.Code696.170

† Close contacts were defined as persons who had prolonged indoor exposure and were in close proximity to a person with pulmonary TB.

§ There are several CDC-recommended LTBI treatment regimens, which range from 3–9 months; seven contacts with LTBI were prescribed 4 months of rifampin, and one contact was prescribed 6 months of isoniazid.

¶ CDC recommends retesting ≥8 weeks after most recent exposure for all contacts who received negative test results before 8 weeks.

** These seven persons were not previously known to have LTBI; they might have previously received testing and did not recall, or they had not seen a provider and did not receive testing.
